# LnCeVar: a comprehensive database of genomic variations that disturb ceRNA network regulation

**DOI:** 10.1093/nar/gkz887

**Published:** 2019-10-16

**Authors:** Peng Wang, Xin Li, Yue Gao, Qiuyan Guo, Shangwei Ning, Yunpeng Zhang, Shipeng Shang, Junwei Wang, Yanxia Wang, Hui Zhi, Ying Fang, Weitao Shen, Guangmei Zhang, Steven Xi Chen, Xia Li

**Affiliations:** 1 College of Bioinformatics Science and Technology, Harbin Medical University, Harbin 150081, China; 2 Department of Public Health Sciences, University of Miami Miller School of Medicine, Miami, FL 33136, USA; 3 Sylvester Comprehensive Cancer Center, University of Miami Miller School of Medicine, Miami, FL 33136, USA; 4 Department of Gynecology, the First Affiliated Hospital of Harbin Medical University, Harbin 150081, China

## Abstract

LnCeVar (http://www.bio-bigdata.net/LnCeVar/) is a comprehensive database that aims to provide genomic variations that disturb lncRNA-associated competing endogenous RNA (ceRNA) network regulation curated from the published literature and high-throughput data sets. LnCeVar curated 119 501 variation–ceRNA events from thousands of samples and cell lines, including: (i) more than 2000 experimentally supported circulating, drug-resistant and prognosis-related lncRNA biomarkers; (ii) 11 418 somatic mutation–ceRNA events from TCGA and COSMIC; (iii) 112 674 CNV–ceRNA events from TCGA; (iv) 67 066 SNP–ceRNA events from the 1000 Genomes Project. LnCeVar provides a user-friendly searching and browsing interface. In addition, as an important supplement of the database, several flexible tools have been developed to aid retrieval and analysis of the data. The LnCeVar–BLAST interface is a convenient way for users to search ceRNAs by interesting sequences. LnCeVar–Function is a tool for performing functional enrichment analysis. LnCeVar–Hallmark identifies dysregulated cancer hallmarks of variation–ceRNA events. LnCeVar–Survival performs COX regression analyses and produces survival curves for variation–ceRNA events. LnCeVar–Network identifies and creates a visualization of dysregulated variation–ceRNA networks. Collectively, LnCeVar will serve as an important resource for investigating the functions and mechanisms of personalized genomic variations that disturb ceRNA network regulation in human diseases.

## INTRODUCTION

Emerging evidence suggests that long noncoding RNAs (lncRNAs) can function as competing endogenous RNAs (ceRNAs) and dynamically buffer the expression of downstream genes during different physiological and pathological processes (1,2). Genomic variations such as single-nucleotide polymorphisms (SNPs) and somatic mutations on lncRNAs will alter the microRNA (miRNA) binding sites and further lead to the gain or loss of ceRNA interactions (3,4). For example, a functional lncRNA HOTAIR genetic variant, rs920778, contributes to gastric cancer susceptibility by disturbing the miR-331-3p binding site (5). The lncRNA linc-RoR functions as a sponge of miR-145-5p, protecting OCT4, SOX2 and NANOG genes from miR-145-mediated suppression. However, this regulation was abolished by an engineered mutation in the miR-145-5p binding sites of linc-RoR (6). Furthermore, copy number variations (CNVs) such as amplifications and deletions will generate or delete large numbers of miRNA-binding sites and also cause disturbances of ceRNA networks (7). By sharing common miRNA-binding sites, the noncoding gene PTENP1 is biologically active in regulating the cellular levels of PTEN. Copy number deletion at the PTENP1 locus can decrease the expression level of PTEN and increase proliferation of cancer cells in a dose-dependent manner, suggesting it could be considered a tumor suppressor gene in colon cancer (8). Chiu *et al.* have performed in-depth evaluation of the lncRNA CNV effects on downstream targets. This work identified hundreds of candidate cancer CNV-lncRNA-target events and provided a resource for studying cancer lncRNAs and their potential roles as onco- and tumor-suppressor genes (9). Identification of these personalized variation events will help us understand individual disease pathology and further contribute to precision medicine.

Recent advances in high-throughput technologies have made it possible to identify individual genomic variations with different molecular and functional phenotypes (10). To date, several databases have been developed to collect genomic variations and their effects on lncRNAs. SomamiR 2.0 is a database of cancer somatic mutations altering ceRNA interactions (4). LncRNASNP2 provides comprehensive information on SNPs and mutations in lncRNAs, as well as their impacts on lncRNA structure and function (11). LncVar is a database of genetic variations associated with long noncoding genes in different species (12). These databases are valuable resources for studying genomic variations and their effects on lncRNAs. However, most of them predict variation–ceRNA interactions only through the gain or loss of miRNA targets according to differing variation status. An analysis integrating both genomic status and changes in ceRNA expression level needs to be undertaken systematically to further understand disease progression. A recent study has used a multivariate multiple regression model to identify SNP–ceRNA interactions and evaluate the effects of variations on ceRNA expression change (3). A limitation of this work is that only coding gene-associated ceRNAs were analyzed. Thus far, to our knowledge, no other specialized resource has been devoted to collecting and analyzing experimentally supported genomic variation–ceRNA interactions, as well as comprehensive annotations.

To meet this need, we describe a comprehensive database, LnCeVar, which documents personalized lncRNA–variation–ceRNA events of high quality manual curation from the published literature and high-throughput identification from individual genomics data. LnCeVar provides 119 501 variation-ceRNA events, including SNPs and CNVs as well as somatic mutations, from thousands of samples and cell lines. More than 2000 experimentally supported circulating, drug-resistant and prognosis-related lncRNA biomarkers were manually curated from the published literature. Expression profiles of ceRNAs and individual clinical information from >10 000 samples from TCGA, COSMIC and 1000 Genomes Project have been integrated into LnCeVar. LnCeVar provides a well-designed, user-friendly interface to query, download and analyze data. In particular, several flexible online tools have been developed to facilitate data analysis and visualization. LnCeVar also provides an illustration of functions, networks, hallmarks and survival information that were disturbed by genomic variations. Collectively, we expect the LnCeVar database to facilitate the identification and analysis of genomic variations that disturb lncRNA-associated ceRNA regulation of individual disease pathology and further contribute to precision medicine. All of the information in LnCeVar is freely available at http://www.bio-bigdata.net/LnCeVar.

## DATA COLLECTION AND DATABASE CONTENT

### Collection of experimentally supported lncRNA–variation–ceRNA events and biomarkers

**Figure 1.**
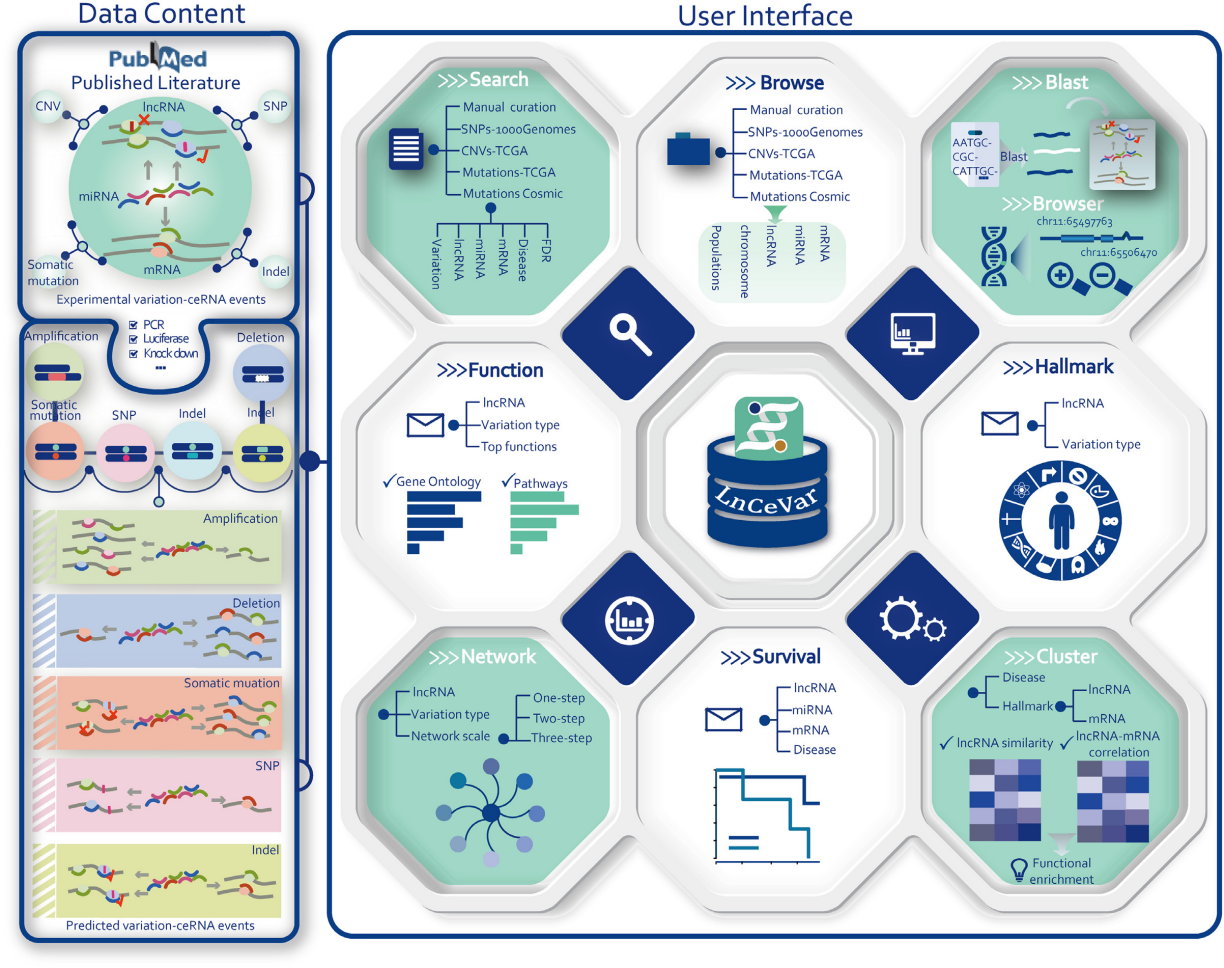
Content and interface of LnCeVar. The left panel contains the database content, which includes lncRNA–variation–ceRNA events identified from the literature and high-throughput experiments. The right panel contains the user interface of LnCeVar. In this panel, the Search, Browse, Blast and Browser interfaces provide flexible ways to query the data set. Online tools, including LnCeVar–Function, LnCeVar–Hallmark, LnCeVar–Network, LnCeVar–Survival and LnCeVar-Cluster, have been developed to perform customized analysis and data visualization.

High confidence variation–ceRNA associations and lncRNA biomarkers were manually curated from the literature and integrated into the LnCeVar database (Figure 1). We retrieved the published literature from PubMed by employing key words related to SNPs, somatic mutations, CNVs and ceRNAs and found >2000 relevant articles (before July 2019, Supplementary Methods). The experimentally supported lncRNA–variation–ceRNA events were manually curated from these papers by at least two researchers. Further, biomarker information was collected if the lncRNA had been experimentally verified to be related to circulating, drug-resistant or prognostic processes. In this work, we manually collected experimentally supported variation–ceRNA associations and biological biomarkers through several steps as previously described (2,13). Only data sets supported by information from high confidence experiments, such as PCR, western blot or the luciferase reporter assay, and other reliable methods, were considered and curated. The exact procedures relating to the experimental types, curation process and criteria used in our pipeline are described in Supplementary Methods. Further, we reviewed the literature by year and found that the number of related works had significantly increased in recent years, and especially in 2018–2019 (Supplementary Figure S1A and B). The rapidly growing number of genomic variation–ceRNA relations and lncRNA biomarkers indicates an urgent need to collect corresponding data sets and develop the LnCeVar database. Currently, LnCeVar documents a total of 2499 experimentally supported variation–ceRNA events and lncRNA biomarkers related to SNPs, somatic mutations, CNVs and circulating, drug-resistant and prognostic processes.

### Identification of lncRNA–variation–ceRNA events from high-throughput data

To provide a comprehensive set of genomic variations on lncRNAs, we collected SNPs, somatic mutations and CNV information from different data sources (Figure 1). SNP genotype data and RNA-sequencing expression data from 432 unrelated human samples from the CEPH (CEU), Finns (FIN), British (GBR), Toscani (TSI) and Yoruba (YRI) populations were downloaded from the 1000 Genomes Project (14). Cancer somatic mutations were collected from COSMIC (15) and TCGA (16). For CNV information, we used GISTIC v2.0 to evaluate the CNV values in TCGA cancer samples (17). LncRNAs located in the wide peak regions identified by GISTIC v2.0 were collected and given a CNV score. LncRNA transcripts were downloaded from GENCODE (v29, GRCH38) and mature miRNA sequences were downloaded from miRBase (v21). We used miRanda (v2010) (18), TargetScan (v.6.0) (19) and RNAhybrid (v.2.1.2) (20) to identify miRNA–lncRNA interactions and evaluate the effects of genomic variations on the miRNA-binding sites. A functional variation was identified if the different genotypes of a variation could change the miRNA–lncRNA interaction (gain, loss or alternative score). The miRNA–mRNA regulations that were validated by strong experimental methods such as luciferase reporter assay, PCR and western blot were derived from TarBase (v8) (21) and miRTarBase (v2018) (22). If a lncRNA and a mRNA interacted with the same miRNA, this lncRNA–miRNA–mRNA competing triplet was termed a candidate ceRNA interaction. To further identify functional lncRNA–variation–ceRNA events, we used a multivariate multiple regression model to investigate whether a given variation regulates the expression of the host lncRNA and downstream competing mRNA (3). In this model, the lncRNA hosting a variation and the downstream mRNA were termed the driver and target gene, respectively. The variation effect for the lncRNA and mRNA could be estimated by the corresponding coefficients on the genotype, and the significance of the multivariate multiple regression model can be assessed by Pillai’s trace test statistics (FDR < 0.05, details in Supplementary Methods, Supplementary Figures S2–S4). Ultimately, 11 418 somatic mutation–ceRNA events from TCGA and COSMIC, 112 674 CNV–ceRNA events from TCGA and 67 066 SNP–ceRNA events from 1000 Genomes Project were identified by LnCeVar.

### Comprehensive annotation of lncRNA–variation–ceRNA events

To facilitate the study of lncRNA–variation–ceRNA events, LnCeVar provides comprehensive annotations of miRNA-binding sites, functions, hallmarks, networks, prognosis, genomic variations etc. For each lncRNA–variation–ceRNA entry, LnCeVar provides a detailed illustration of miRNA-binding status (gain, loss or alternative score) that was disturbed by genomic variations on the lncRNA sequence. A ‘guilt-by-association’ strategy (2) has been adopted in LnCeVar to infer the biological functions, pathways and cancer hallmarks that are dysregulated by genomic variations (Supplementary Methods). Currently, several functional contexts have been integrated into LnCeVar to provide a comprehensive annotation background. A total of 5917 gene sets representing functional terms were collected from Gene Ontology (23). A total of 1329 biological pathways including KEGG (24), Reactome (25) etc. were collected from MSigDB (26). We manually curated gene sets of the ten cancer hallmark processes that have been determined to promote tumor growth and metastasis (27). Gene sets from corresponding GO terms were mapped to each of the cancer hallmarks (28). For each lncRNA, LnCeVar constructs a dysregulated ceRNA network and provides a graphic illustration showing the ceRNA interactions that were disturbed by genomic variations. In survival analysis, LnCeVar collects clinical follow-up information on 10 141 patients from TCGA and further builds a risk score model based on the linear combination of ceRNA expression values weighted by the Cox regression coefficient (29). A Kaplan–Meier survival analysis is performed for the two groups of patients (divided by median or mean risk score), and statistical significance is assessed by log-rank test (*P* < 0.05). To provide functional support of genomic variations, annotations such as OMIM (30), COSMIC and dbSNP have been integrated into LnCeVar.

### A panel of tools for data discovery and analysis

With the development of high-throughput technologies, the fast growing number of noncoding RNAs and genomic variations urgently need to be analyzed to facilitate disease pathology dissection and cancer biomarker discovery. To meet this end, several flexible tools have been developed to retrieve and analyze the data (Figure 1). The LnCeVar-BLAST tool is a convenient way for users to query the database by interesting sequences. The LnCeVar-Browser is a web-based genome browser that dynamically displays different tracks, including reference sequences, genomic variations, ceRNA transcripts and miRNA-binding sites. The LnCeVar-Cluster tool provides cluster profiles between different lncRNAs and ceRNAs. LnCeVar-Function performs functional enrichment analysis. LnCeVar-Hallmark identifies dysregulated cancer hallmarks associated with variation–ceRNA events. LnCeVar–Survival performs a COX regression analysis and produces survival curves for variation–ceRNA events. LnCeVar-Network identifies and visualizes dysregulated variation–ceRNA networks.

## DATABASE CONSTRUCTION AND USER INTERFACE

### Flexible ways to access the data set

The LnCeVar online web server was developed using Java Server Pages within Tomcat software (v6). All data sets were documented and managed in MySQL (v 5.5) data server. LnCeVar provides user-friendly interfaces and flexible routes for data access and discovery. (i) A quick search interface has been developed in the home page that allows users to search both experimentally supported and predicted data sets. The inputted key words can be any genomic variations (SNPs, mutations and CNVs), ceRNAs (lncRNAs, miRNAs and mRNAs), diseases, cell lines, cytobands, primary sites etc. The global search engine will list all potential results matching the key words. (ii) The Browse page provides a catalog allowing users to browse the LnCeVar database according to different categories. (iii) Because of the fast growing number of newly identified RNA sequences, a data query tool named LnCeVar-BLAST has been developed to implement a customized sequence search. Users can input new RNA sequences to identify related lncRNA–variation–ceRNA events. (iv) LnCeVar-Browser is a web-based genome browser that dynamically displays different tracks for genomic variations and ceRNAs. It provides comprehensive tracks including reference sequences, transcripts, miRNA-binding sites (identified by the target prediction method and high-throughput CLIP-Seq peaks). (v) The customized Results table can be flexibly downloaded by clicking the ‘Copy,’ ‘Excel,’ ‘CSV’ and ‘PDF’ buttons through all querying steps. In addition, all associated data sets can be freely accessed in the Download page.

### An example of LnCeVar use

**Figure 2.**
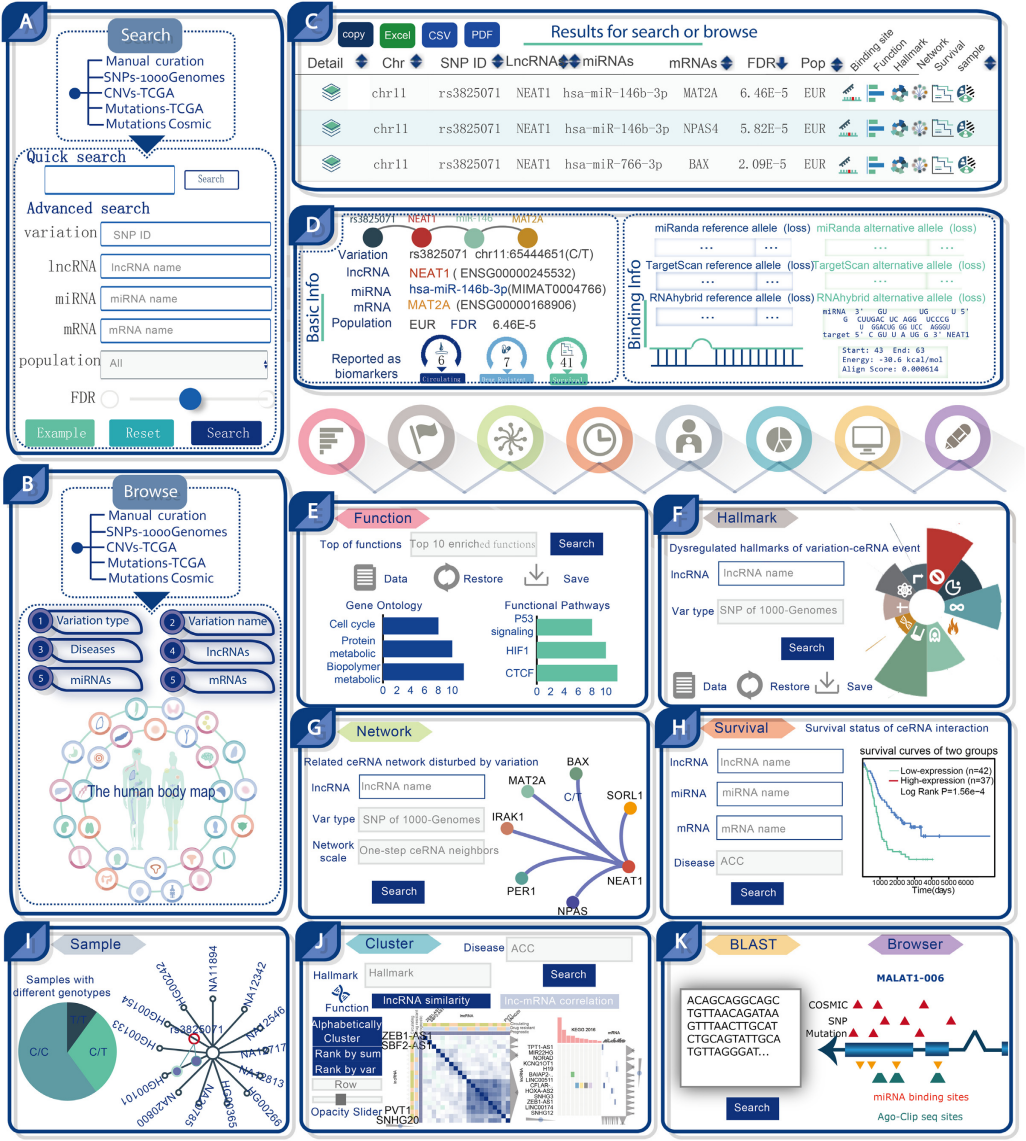
Workflow and an example of using LnCeVar. (**A**) Interface of the Search module using the example of NEAT1. (**B**) Interfaces of the Browse module and human body map. (**C**) Data table resulting from a search and browse for NEAT1. (**D**) Basic information and miRNA-binding status for NEAT1–variation–ceRNA events. (**E**) Functional analysis of NEAT1 based on the context of GO terms and biological pathways. (**F**) Hallmark analysis of NEAT1 based on related biological processes. (**G**) Global view of all possible NEAT1-related ceRNA interactions disturbed by genomic variations. (**H**) Survival analysis and Kaplan–Meier survival curves for NEAT1-associating ceRNAs. (**I**) Distribution of genomic variations on NEAT1 in different samples and populations. (**J**) Cluster profiles of NEAT1 and associated ceRNAs. (**K**) LnCeVar–BLAST and LnCeVar–Browser interfaces for customized sequence search and genomic search, respectively.

As an example, lncRNA NEAT1 was used as an input in the Quick search and Advanced search interface (Figure 2A). In addition, a Browse page was designed for general perusal of the database based on different data sets, and a human disease body map was provided to show the LnCeVar statistics (Figure 2B). According to the inputs, all possible lncRNA–variation–ceRNA events will be listed in the Results page (Figure 2C). To filter out interesting results, users can reorder the data table by clicking on the table headers of different columns. The first and last columns (Figure 2C) will directly lead users to the detailed information and analyzing tools of each record (Figure 2D). In the detailed information page, LnCeVar provides basic information on NEAT1–variation–ceRNA events, as well as whether NEAT1 has been reported as a circulating biological biomarker associated with drug resistance or survival. Detailed information on miRNA-binding sites (gain, loss or alternative score) that were disturbed by genomic variations on NEAT1 is also provided (Figure 2D). To further analyze the data set, several sections have been provided and can be easily accessed via the navigation bar on top of the page (Figure 2E–I). The Functions and Hallmarks sections perform functional analyses based on molecular pathways and biological processes (Figure 2E and F). The Network section provides a global view of all possible NEAT1-related ceRNA interactions disturbed by genomic variations (Figure 2G). The Survival section performs a COX regression analysis and provides Kaplan–Meier survival curves for all competing members (lncRNA, miRNA and mRNA) and the ceRNA interaction (Figure 2H). The Samples section provides a pie graph and a genotype tree to illustrate the distribution of genomic variations on NEAT1 in different samples and populations (Figure 2I). To further facilitate data analysis and discovery, the LnCeVar–Cluster interface provides similarity profiles between NEAT1 and other lncRNAs and a cluster profile of NEAT1-associated ceRNAs (Figure 2J). And the LnCeVar–BLAST and LnCeVar–Browser interfaces have been developed to implement a customized sequence search and genomic search, respectively (Figure 2K).

## CONCLUSIONS AND FUTURE DEVELOPMENT

With the development of high-throughput technology and experimental validation methods, the numbers of lncRNA–variation–ceRNA events and disease biomarkers have significantly increased in recent years, and especially in 2018–2019 (Supplementary Figure S1). Exhaustive sequencing of cancer genomes to yield complete catalogs of all classes of genomic variations will gather pace over the next few years. Based on the biological big data, the predicted genomic variations such as SNPs and CNVs may not be functional. Most predictions are used with little support for functional relevance. The fast growing high-throughput technology and studies reveal an urgent need to collect corresponding data sets and to provide comprehensive annotations for individual genomic variations. To meet this need, we describe a comprehensive database, LnCeVar, which documents personalized lncRNA–variation–ceRNA events of high-quality manual curation from the published literature and high-throughput identification from individual genomics data. The current database offers insights into the complexity of personalized genomic variations that will disturb ceRNA regulation in human diseases, and demonstrates the potential for the generation of active biomarkers that may be used to uncover individual disease pathology and further contribute to precision medicine. A comparison between LnCeVar and other databases in terms of data source and characteristics was shown in Supplementary Table S1. We expect that the number of lncRNA–variation–ceRNA events identified from high-confidence experiments or high-throughput analyses will continue to increase rapidly in future releases of the LnCeVar database. In the ceRNA regulation network, genomic variations on other components (mRNAs and miRNAs) also have disturbing effects of ceRNA networks. In our previous work, we have developed a manually curated database that provides comprehensive experimentally supported associations among SNPs, miRNAs and human diseases (31). A recent study has used a multivariate multiple regression model to identify SNPs on mRNAs and evaluate the effects of variations on ceRNA expression change (3). In the future work, we will improve the LnCeVar database by adding the other components such as miRNAs and mRNAs that disturb ceRNA network regulation in human diseases. We will continually maintain and update the LnCeVar database with the latest version of data sets and methods. More and more data sets and functional web-based tools will be integrated into LnCeVar, which will facilitate and improve our understanding of the individualized mechanisms behind disease pathology.

## Supplementary Material

gkz887_Supplemental_File
