# Supplementary material for: LnCeVar: a comprehensive database of genomic variations that disturb ceRNA network regulation
Source: Nucleic Acids Res. 2019 Oct 16;48(D1):D111–7. doi: 10.1093/nar/gkz887 (PMC7145649; doi:10.1093/nar/gkz887)
Supplement: gkz887_Supplemental_File [file gkz887_supplemental_file.pdf]

Supplementary Methods

Manual curation of experimentally supported lncRNA-variation-ceRNA events and biomarkers

To collect high-confidence variation-ceRNA associations and lncRNA biomarkers, we retrieved published literature from PubMed related to SNPs, somatic mutations, CNVs and ceRNAs. We used the following combination of key words "(miRNA sponge OR ceRNA OR miRNA decoy OR competing RNA OR antagomir OR miRNA mediated) AND (SNP OR mutation OR CNV)" to search the PubMed database and found more than 2,000 relevant articles. The experimentally supported lncRNA-variation-ceRNA events were manually curated from these published articles by at least two researchers. Further, we used the following combination of key words "(circulating OR drug resistant OR prognostic) AND lncRNA" to collect biomarker records. The biomarker information was collected if an lncRNA had been experimentally verified to be related to a circulating, drug-resistant or prognostic process. In this work, we manually collected experimentally supported variation-ceRNA associations and biological biomarkers through several steps as previously described (1,2). Only datasets supported by information from high confidence experiments, such as PCR, western blot or luciferase reporter assay, and other reliable methods were considered and curated.

Identification of miRNA-target regulation

LncRNA transcripts were downloaded from GENCODE (v29, GRCH38) and mature miRNA sequences were downloaded from miRBase (v21). We used different methods to identify miRNA-target relationships and evaluate the effects of genomic variations on the miRNA

binding sites. The miRNA-lncRNA interactions were predicted using three miRNA target prediction methods: miRanda (v2010) (3), TargetScan (v.6.0) (4) and RNAhybrid (v.2.1.2) (5) with strict thresholds (miRanda: score >160 and energy <-20; TargetScan: context score<-0.4; RNAhybrid: mfe <-25 and P<0.01). A functional variation was identified if the different genotypes of a variation could change the miRNA-lncRNA interaction (gain, loss or alternative score). The miRNA-mRNA regulations that were validated by strong experimental methods, such as luciferase reporter assay, PCR and western blot, were derived from TarBase (v8) (6) and miRTarBase (v2018) (7). If the lncRNA and mRNA interacted with the same miRNA, the lncRNA-miRNA-mRNA competing triplet was termed a candidate ceRNA interaction. To further identify functional lncRNA-variation-ceRNA events, we used a multivariate multiple regression model to investigate whether a given variation regulated the expression of the host lncRNA and downstream competing mRNA (see below).

Identification of lncRNA-SNP-ceRNA events

For each lncRNA-SNP-ceRNA unit, we applied a multivariate multiple regression model to explore whether a given SNP could produce or alter the status of some ceRNA relationship (Figure S2A). So, for the potential competing lncRNA (Y_l) and mRNA (Y_m) pair, we included predictors that might have an effect on their expression levels. In particular, they were genotypes of variants across samples (G), the residual of the miRNA expression (M_r) value calculated by PEER software (8), the PEER factor of the lncRNA expression level (PF_l), the PEER factor of the mRNA expression level (PF_m) and the first three principal components derived from an individual's genotype. The variants used for principal component analysis were

filtered according to a previous study (9) and computed using EIGENSTRAT(10). The detailed model was designed as:

$$(Y_l, Y_m) = \mathrm{G} + M_r + PF_l + PF_m + PC + \varepsilon$$

where ε represents the error vector $(\varepsilon_1, \varepsilon_2)'$ and is assumed to follow a Gaussian distribution. The PEER factor can correct known technical and biological covariates in the expression profile (9,11); to avoid overfitting, we chose to use 10 PEER factors in each separate population, and to use 30 PEER factors in the combined population (Figure S2B-C). So, for each analyzed lncRNA-SNP-ceRNA, we can estimate the effect of G on Y_l and Y_m (referred as β_l and β_m). We tested the significance of the model using Pillai's trace test statistics. Further, we expect the trends of the effects of a certain variant on lncRNA and miRNA are opposite in direction, so we only retained lncRNA-SNP-ceRNA units with $\beta_l \times \beta_m < 0$ and false discovery rate (FDR) < 0.05.

Identification of lncRNA-CNV-ceRNA events

Similar to the lncRNA-SNP-ceRNA identification process, we also identified CNV-mediated ceRNA units using a multivariate multiple regression model (Figure S3A). We propose if there exists a CNV in the lncRNA region, it can have an effect on lncRNA expression and alter the competing status of lncRNA and mRNA. So for any lncRNA (Y_l) and mRNA (Y_m) competing pair, we sought to investigate the effect of the CNV level (C) of the lncRNA; also, we corrected the miRNA expression (M_r) effect, the PEER factor of lncRNA (PF_l), mRNA (PF_m) and CNV (PF_C). The regression model is as follows:

$$(Y_l, Y_m) = \mathrm{C} + M_r + PF_l + PF_m + PF_C + \varepsilon$$

Five PEER factors were used for cancers with samples between 20 and 50, 10 PEER factors were used for cancers with samples between 50 and 100, 15 PEER factors were selected for cancers with samples between 100 and 550 (Figure S3B-D). Based on the model, we could obtain the CNV effect on lncRNA and mRNA (β_l and β_m), and we expect CNV can have effects on both in the same direction. Thus, in the lncRNA-CNV-ceRNA identification section, we only retained events with $\beta_l \times \beta_m > 0$ and false discovery rate (FDR) < 0.05.

Identification of lncRNA-mutation-ceRNA events

For somatic mutations detected in TCGA and COSMIC samples, we identified mutations located in lncRNA regions that can affect the binding affinity between the mutant and normal reference alleles. Then we mapped them into lncRNA-miRNA-mRNA competing triplets to form mutation-ceRNA events (Figure S3). In this step, the lncRNA-miRNA-mRNA competing triplets were downloaded from the LncACTdb 2.0 database (2).

Performance of functional analysis based on ceRNA theory

LnCeVar develops the LnCeVar-Function and LnCeVar-Hallmark tools to perform functional analysis of lncRNAs based on a “guilt-by-association” strategy. For a lncRNA, the corresponding downstream mRNA targets in the lncRNA-variation-ceRNA events were used to perform a function enrichment analysis. LnCeVar-Function collected thousands of pathways and biological processes as functional context. For pathway annotation, a total of 1,329 pathway gene sets derived from KEGG, BioCarta, Reactome, Pathway Interaction Database

and other biological pathway databases were collected as functional background. For Gene Ontology annotation, a total of 5,917 gene sets representing functional terms were collected. We manually curated gene sets of the ten cancer hallmark processes, which have been determined to promote tumor growth and metastasis (12). Gene sets from corresponding GO terms were mapped to each of the cancer hallmarks (13). LnCeVar performs a hypergeometric test to evaluate the enrichment significance based on different functional contexts. If there are a total of *N* genes in the genome, of which *S* are involved in the gene set under investigation, and there are a total of *M* interesting target genes for analysis, of which *x* are involved in the same function gene set, then the P value can be calculated as:

$$P = 1 - \sum_{t=0}^{x} \frac{\binom{S}{t}\binom{N-S}{M-t}}{\binom{N}{M}}$$

Significantly enriched functions were defined at the P<0.05 level and further illustrated as a bar graph based on –log 10 transformed P values.

Survival analysis of ceRNAs

The LnCeVar-Survival tool performs a COX regression analysis and provides Kaplan-Meier survival curves for each competing member (lncRNA, miRNA and mRNA) and the whole ceRNA interaction. Clinical follow-up information on 10,141 patients from TCGA was collected to carry out the survival analysis. LnCeVar performs a univariate Cox regression analysis to evaluate the association between survival and the expression level of each lncRNA-miRNA-mRNA member in a ceRNA interaction. A risk score model was constructed to evaluate the association between survival and expression in a certain disease, which takes into account both the strength and positive/negative association between each competing RNA and

probability of survival (2). For each sample, the risk score was calculated by linearly combining the ceRNA expression values weighted by the Cox regression coefficients:

$$Risk\ score = \sum_{i=1}^{n} \beta_i Exp(c_i)$$

where β_i is the Cox regression coefficient of a lncRNA, miRNA or mRNA in a ceRNA interaction (indicated as c_i), *n* is the number of competing RNAs (defined as 3) and $Exp(c_i)$ is the expression value of competing RNA c_i in the corresponding sample. The median and mean risk scores were used as cut-off points to divide samples into high and low-risk groups.

Construction of ceRNA networks disturbed by genomic variations

The LnCeVar-Network tool provides a global view of all possible related ceRNAs interactions disturbed by genomic variations. For each lncRNA-variation-ceRNA entry, LnCeVar constructs a network and further provides a graphic illustration consisting of this ceRNA interaction and its associated competing neighbors. The network can be shown at different scales by adjusting the parameters of different neighbours. For the one-step-neighbours scale, the top 20 ceRNA interactions that were disturbed by genomic variations (ordered by the FDR value) of the lncRNA are illustrated. For the two-step-neighbours and three-step-neighbours scales, this network will expand to another 20 and 40 ceRNA interactions that were disturbed by genomic variations.

Implementation of the LnCeVar-BLAST interface

The LnCeVar-BLAST interface is a convenient way for users to query the dataset by inputting custom sequences. To compare an inputted sequence to the LnCeVar database, the Basic

Local Alignment Search Tool (BLAST, https://blast.ncbi.nlm.nih.gov/Blast.cgi) is implemented and used to calculate the statistical significance of matches. LnCeVar set the matching parameters as -perc_identity 80 -outfmt “7 qacc sacc evalue length pident.” Both lncRNA and mRNA transcripts that have high similarity (>80% identity) with the inputted sequence would be listed in a Results table.

References

1. Gao, Y., Wang, P., Wang, Y., Ma, X., Zhi, H., Zhou, D., Li, X., Fang, Y., Shen, W., Xu, Y. *et al.* (2019) Lnc2Cancer v2.0: updated database of experimentally supported long non-coding RNAs in human cancers. *Nucleic acids research*, **47**, D1028-D1033.
2. Wang, P., Li, X., Gao, Y., Guo, Q., Wang, Y., Fang, Y., Ma, X., Zhi, H., Zhou, D., Shen, W. *et al.* (2019) LncACTdb 2.0: an updated database of experimentally supported ceRNA interactions curated from low- and high-throughput experiments. *Nucleic acids research*, **47**, D121-D127.
3. Betel, D., Wilson, M., Gabow, A., Marks, D.S. and Sander, C. (2008) The microRNA.org resource: targets and expression. *Nucleic Acids Res*, **36**, D149-153.
4. Friedman, R.C., Farh, K.K., Burge, C.B. and Bartel, D.P. (2009) Most mammalian mRNAs are conserved targets of microRNAs. *Genome Res*, **19**, 92-105.
5. Rehmsmeier, M., Steffen, P., Hochsmann, M. and Giegerich, R. (2004) Fast and effective prediction of microRNA/target duplexes. *Rna*, **10**, 1507-1517.
6. Karagkouni, D., Paraskevopoulou, M.D., Chatzopoulos, S., Vlachos, I.S., Tastsoglou, S., Kanellos, I., Papadimitriou, D., Kavakiotis, I., Maniou, S., Skoufos, G. *et al.* (2018) DIANA-TarBase v8: a decade-long collection of experimentally supported miRNA-gene interactions. *Nucleic acids research*, **46**, D239-D245.
7. Chou, C.H., Shrestha, S., Yang, C.D., Chang, N.W., Lin, Y.L., Liao, K.W., Huang, W.C., Sun, T.H., Tu, S.J., Lee, W.H. *et al.* (2018) miRTarBase update 2018: a resource for experimentally validated microRNA-target interactions. *Nucleic acids research*, **46**, D296-D302.
8. Stegle, O., Parts, L., Piipari, M., Winn, J. and Durbin, R. (2012) Using probabilistic estimation of expression residuals (PEER) to obtain increased power and interpretability of gene expression analyses. *Nature protocols*, **7**, 500-507.
9. Consortium, G.T., Laboratory, D.A., Coordinating Center -Analysis Working, G., Statistical Methods groups-Analysis Working, G., Enhancing, G.g., Fund, N.I.H.C., Nih/Nci, Nih/Nhgri, Nih/Nimh, Nih/Nida *et al.* (2017) Genetic effects on gene expression across human tissues. *Nature*, **550**, 204-213.
10. Price, A.L., Patterson, N.J., Plenge, R.M., Weinblatt, M.E., Shadick, N.A. and Reich, D. (2006) Principal components analysis corrects for stratification in genome-wide association studies. *Nature genetics*, **38**, 904-909.
11. Lappalainen, T., Sammeth, M., Friedlander, M.R., t Hoen, P.A., Monlong, J., Rivas, M.A., Gonzalez-Porta, M., Kurbatova, N., Griebel, T., Ferreira, P.G. *et al.* (2013) Transcriptome and genome sequencing uncovers functional variation in humans. *Nature*, **501**, 506-511.
12. Hanahan, D. and Weinberg, R.A. (2011) Hallmarks of cancer: the next generation. *Cell*, **144**, 646-674.
13. Plaisier, C.L., Pan, M. and Baliga, N.S. (2012) A miRNA-regulatory network explains how dysregulated miRNAs perturb oncogenic processes across diverse cancers. *Genome research*, **22**, 2302-2314.

Supplementary Figures

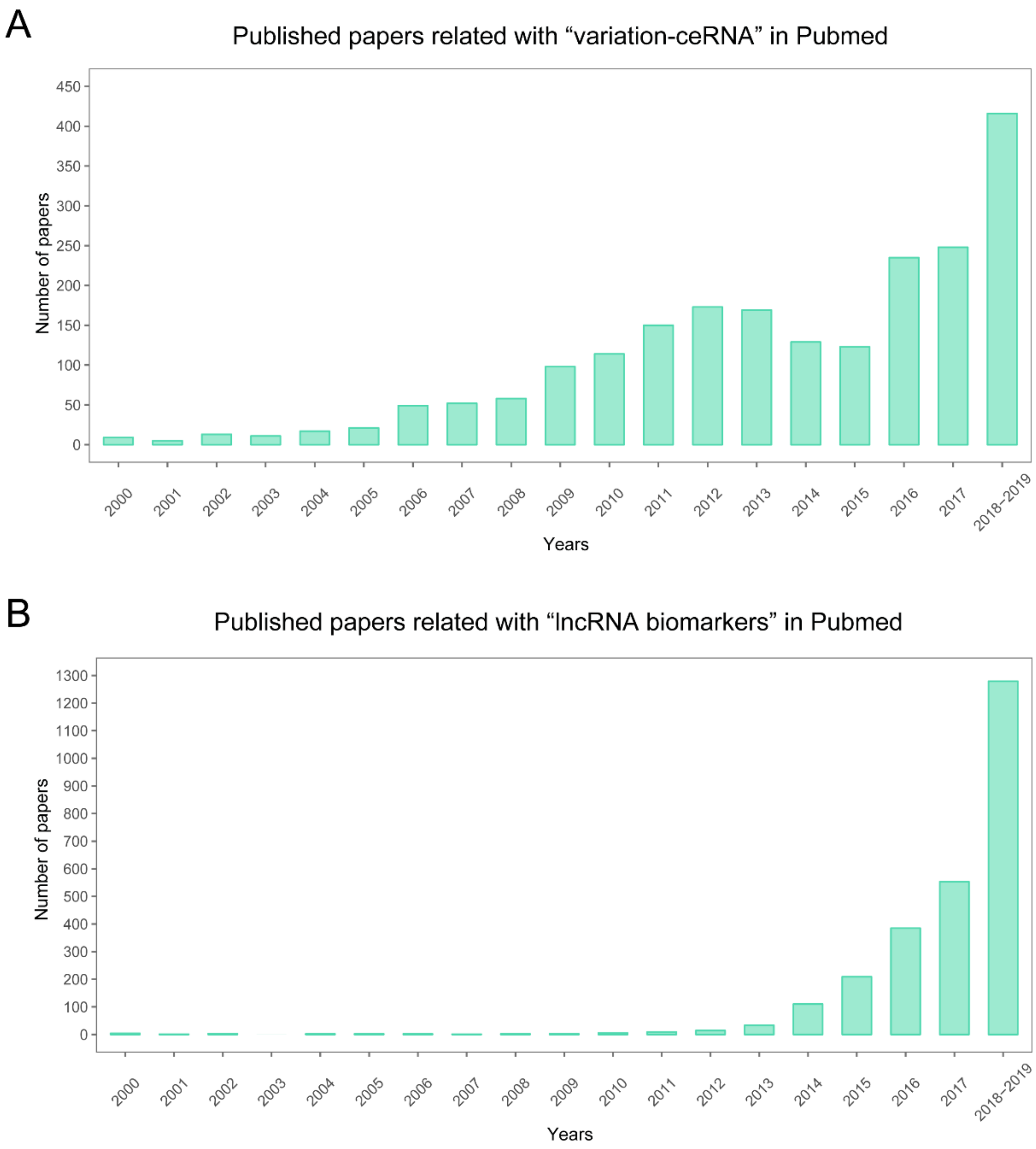

Figure S1. Published literature related to variation-ceRNA and lncRNA biomarkers in recent years. (A) Papers related to variation-ceRNA items. (B) Papers related to lncRNA biomarker items.

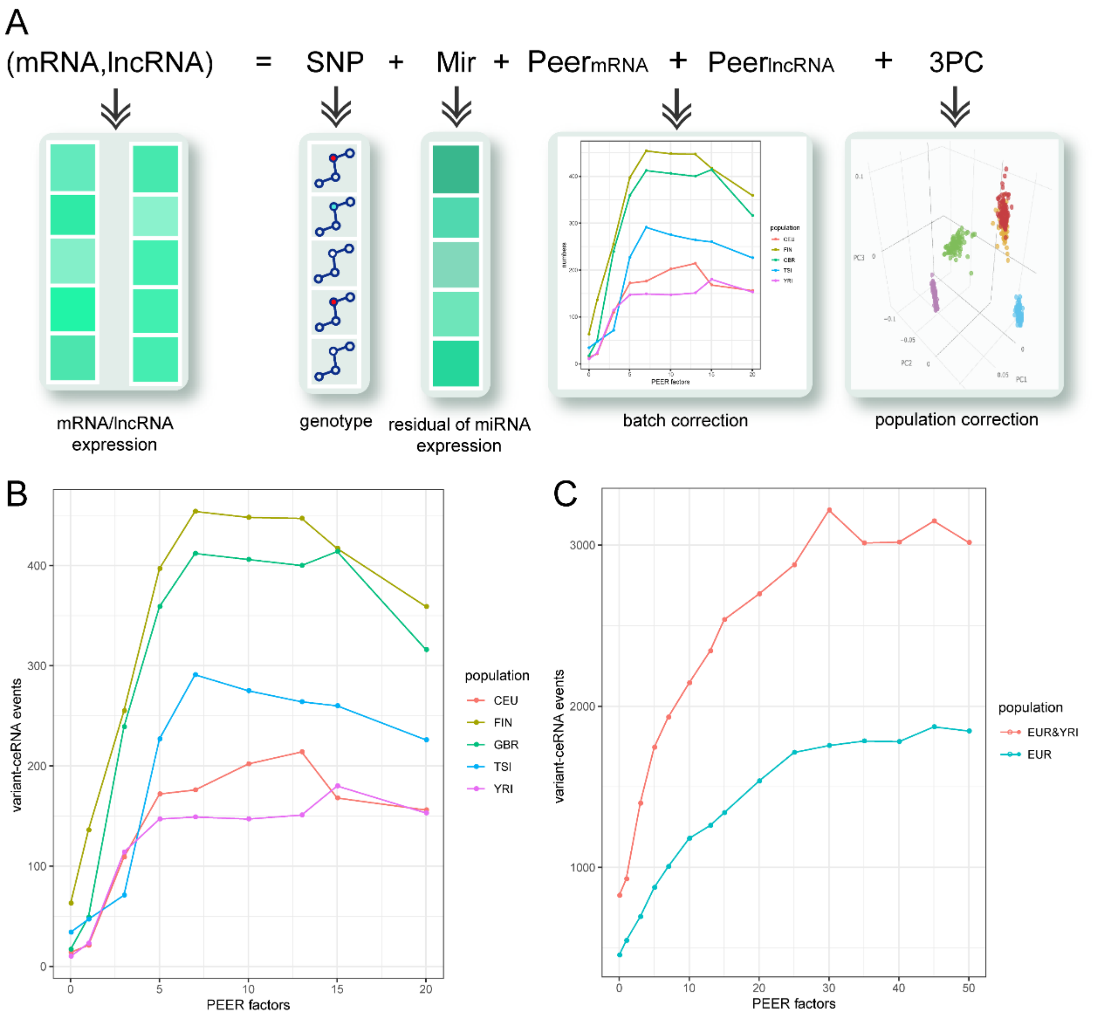

Figure S2. Pipeline to identify lncRNA-SNP-ceRNA events. (A) Illustration of the multivariate multiple regression model. (B) For an independent population (sample number between 80 and 90), we used 10 PEER factors to correct the hidden covariate. (C) For the combined population, EUR (include CEU, FIN, GBR and TSI) and EUR&YRI (include CEU, FIN, GBR, TSI and YRI), we used 30 PEER factors to correct the hidden covariate.

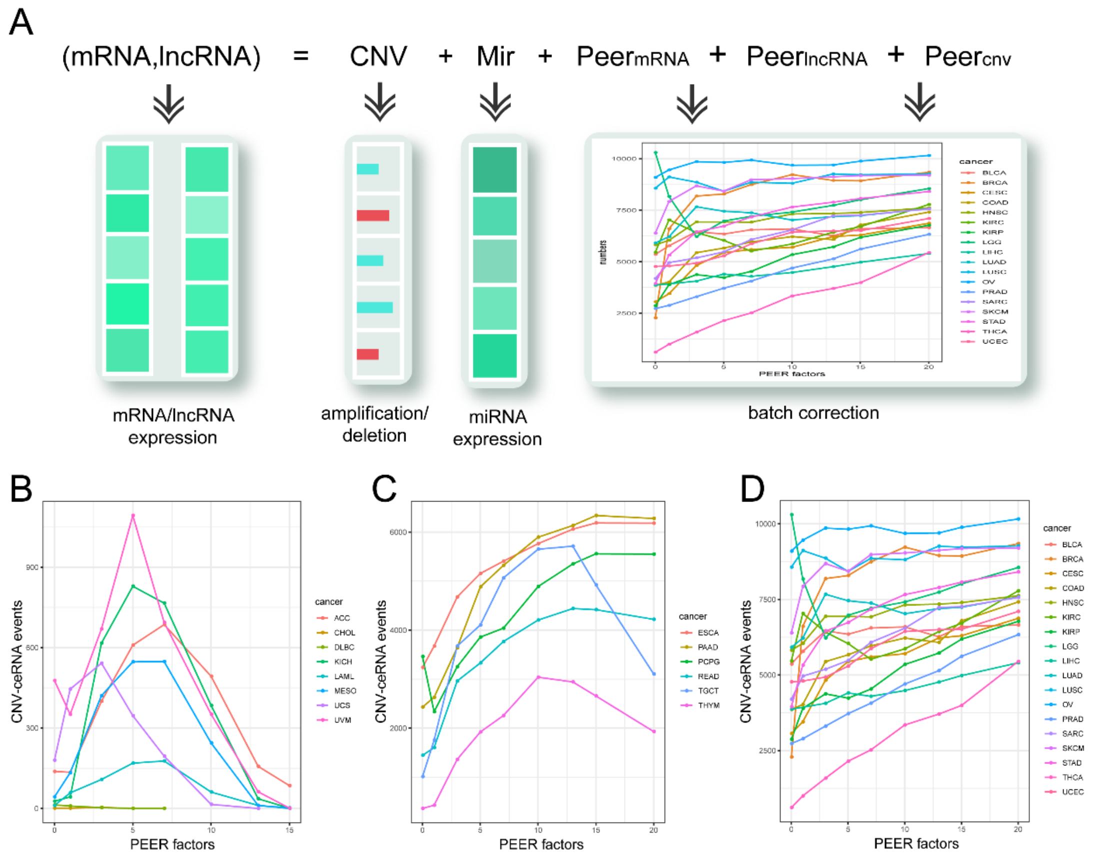

Figure S3. Pipeline to identify lncRNA-CNV-ceRNA events. (A) Illustration of the multivariate multiple regression model. (B) For cancers with a sample number between 20 and 50, we used 5 PEER factors to correct the hidden covariate. (C) For cancers with a sample number between 50 and 100, we selected 10 PEER factors to correct the hidden covariate. (D) For cancers with a sample number between 100 and 550, we selected 15 PEER factors to correct the hidden covariate.

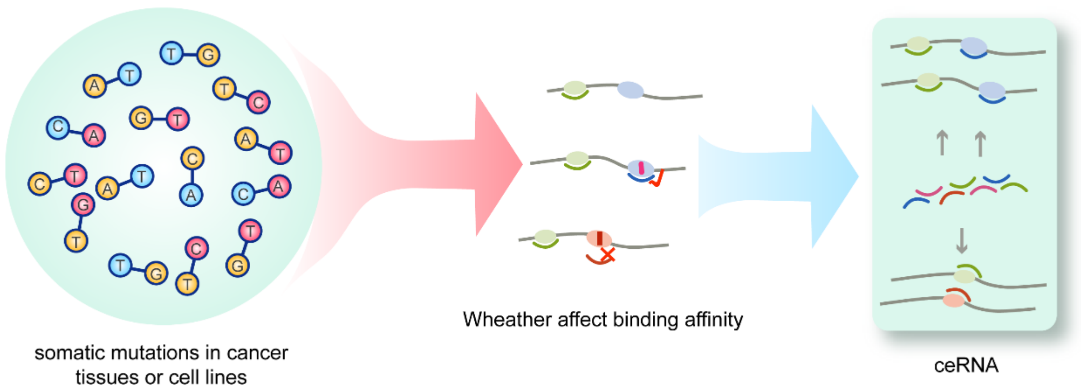

Figure S4. Pipeline to identify lncRNA-mutation-ceRNA events. Somatic mutations located in lncRNA regions that could affect the miRNA binding affinity between the mutant and normal reference alleles were identified. They were mapped into lncRNA-miRNA-mRNA competing triplets to form mutation-ceRNA events.

Supplementary Tables

Table S1. Content and statistics of LnCeVar and other works focusing on genomic variations and lncRNAs.

Datasets and features	LnCeVar	lncRNASNP2	SomamiR 2.0	LncVar
Variation sources	TCGA, COSMIC, 1000 Genomes Project and manual curation	TCGA, COSMIC and GWAS	COSMIC and GWAS	TCGA and dbSNP
Variation types	CNV, SNP and somatic mutation	SNP and somatic mutation	Somatic mutation	CNV and SNP
Variation effects	Altering miRNA target site, multivariate multiple regression and manual curation	FATHMM score and lncRNA structure	Altering miRNA target site	Altering TFBS, m^6A modification, micropeptide and gene fusion
Diseases and Phenotypes	TCGA cancers, COSMIC diseases, manually curated diseases and 1000 Genomes Project normal populations	Diseases from LncRNADisease	COSMIC diseases	TCGA cancers
Functional contexts	Gene Ontology, KEGG, Cancer Hallmarks, BioCarta, Reactome, PID etc.	NA	KEGG	NA
Biomarkers	Prognosis, circulating, and drug-resistant	NA	NA	Prognosis
MiRNA-lncRNA interactions	miRanda, TargetScan and RNAhybrid	miRanda, PITA and TargetScan	TargetScan, CLASH, PAR-CLIP and HITS-CLIP	NA
MiRNA-mRNAs interaction	TarBase and miRTarBase	NA	TargetScan, CLASH, PAR-CLIP and HITS-CLIP	NA
Network analysis	Yes	NA	NA	NA
Survival analysis	Yes	Yes	NA	Yes
Online prediction	Yes	Yes	NA	NA
Genome browser	Yes	NA	Yes	Yes
Species	Human	Human and mouse	Human	8 species
